# Editorial Comment: Training of Brazilian urology residents in laparoscopy: results of a national survey

**DOI:** 10.1590/S1677-5538.IBJU.2018.0668.1

**Published:** 2020-01-10

**Authors:** Mauricio Rubinstein

**Affiliations:** 1 Departamento de Urologia Universidade Federal do Estado do Rio de Janeiro Rio de JaneiroRJ Brasil Departamento de Urologia da Universidade Federal do Estado do Rio de Janeiro - UNIRIO, Rio de Janeiro, RJ, Brasil

Over the last two decades we have seen the development of the minimally invasive surgery (MIS) on the Urology field and laparoscopy was the main part of it, considered as a great option for many of the urological conditions.

The laparoscopic procedures, as in other specialties, became popular by reducing morbidity, convalescence period and showed good results at the literature, becoming attractive for most of urologists.

On this paper, the authors evaluate the access of Brazilian urology residents to laparoscopy surgery, methods of training and perspectives (1).

The Brazilian paper showed a nice response rate (85% of residents) and most of the medical doctors were from the academic hospitals. The most common laparoscopic procedure was radical nephrectomy (73.2%), but less than one third (28.8%) of residents acted as surgeons.

On other nice study about the same issue, surgeons from Portugal compared themselves training with the rest of Europe, showing that all of them had good access to laparoscopic procedures, mostly as assistant (2).

Among the Europeans, the most commonly performed procedure was total nephrectomy also. Most residents rate their motivation to perform laparoscopy in the future as “High” or “Very High”, and plan performing a post-residency fellowship in this field.

On Brazil, 61% of residents did not participate in hands-on courses or fellowship in laparoscopy and almost 30% of them affirms that they are prepared for professional life regarding urologic laparoscopy. A number that is suboptimal for the urology market after the learning period.

Van der Poel et al. (3) also studied the training in minimally invasive surgery at the European Association of Urology. As the Brazilian authors, they showed that the training in MIS has shifted from ‘see-one-do-one-teach-one’ to a structured learning, from e-learning to skills laboratory and modular training settings (4).

The authors concluded that Brazilian urologic residents have access to laparoscopy and actively participate in the learning process and that this should be encouraged.

There is new scenario at the surgery specialties. Robotic surgery has became the new revolution in modern surgery, combining all the benefits of minimally invasive surgery with the advantage of three dimensions (3D) and better intracorporeal instrumentation. It is rapidly expanding in South America, mainly in Brazil, although still very far from most of the hospitals. The Urology staff must be prepared to teach our future residents at this new technology.
